# Cancer cell-derived extracellular vesicles: a potential target for overcoming tumor immunotherapy resistance and immune evasion strategies

**DOI:** 10.3389/fimmu.2025.1601266

**Published:** 2025-06-12

**Authors:** Minseo Ahn, Jeong-Geon Mun, Yohan Han, Jae Ho Seo

**Affiliations:** ^1^ Department of Biochemistry, Wonkwang University School of Medicine, Iksan, Republic of Korea; ^2^ Sarcopenia Total Solution Center, Wonkwang University School of Medicine, Iksan, Republic of Korea; ^3^ Department of Oriental Pharmacy, College of Pharmacy, Wonkwang-Oriental Medicines Research Institute, Wonkwang University, Iksan, Republic of Korea; ^4^ Department of Microbiology, Wonkwang University School of Medicine, Iksan, Republic of Korea; ^5^ Institute of Wonkwang Medical Science, Wonkwang University School of Medicine, Iksan, Republic of Korea

**Keywords:** extracellular vesicles, tumor microenvironment, cancer therapy, immunotherapeutic resistance, immune checkpoint inhibitors, immune cells

## Abstract

Extracellular vesicles (EVs), including exosomes and microvesicles, play crucial roles in cancer progression by mediating the communication between cancer cells and their microenvironment. Cancer cell-derived EVs promote tumor growth, metastasis, and immune evasion by carrying bioactive materials, such as proteins, RNAs, DNA fragments, and lipids but, immunotherapy aims to enhance the immune response against cancer; however, resistance remains a major challenge. Cancer cell-derived EVs contribute to this resistance by delivering immunosuppressive molecules that impair T cell activation, promote the expansion of regulatory T cells (Tregs), and reduce natural killer (NK) cell cytotoxicity, thereby allowing cancer cells to evade immune surveillance. Additionally, cancer cell-derived EVs can carry immune checkpoint proteins, such as Programmed Death-Ligand 1 (PD-L1), which bind to the Programmed Death-1 (PD-1) receptor on T cells, leading to T cell exhaustion and reduced anti-tumor activity. This mechanism reflects how cancer cells directly evade immune detection and contributes to the overall resistance to immune checkpoint blockade therapies, such as anti-PD-1 or anti-PD-L1 antibodies. By delivering these immunomodulatory molecules, EVs not only contribute to local immune suppression but also create a systemic environment that is less favorable for effective anticancer immunity. Therefore, understanding the role of EVs in the immunotherapy resistance is crucial for developing targeted strategies to counteract their effects and ultimately improve therapeutic outcomes. Here we encourage researchers to pay more attention to the role of cancer cell-derived EVs in overcoming immunotherapeutic resistance, because such efforts may be one of the most promising approaches to address immunotherapy resistance in the future.

## 1 Introduction

Extracellular vesicles (EVs) are nanosized, membrane-enclosed vesicles released by nearly all cell types, including cancer cells. These vesicles, mainly classified as exosomes (30–150 nm) and microvesicles (100–1,000 nm), function as intercellular messengers that transport bioactive molecules such as proteins, lipids, RNAs, and DNA fragments. By facilitating the exchange of these components, EVs influence a wide range of physiological and pathological processes, including immune modulation, tissue repair, and disease progression. In cancer, EVs play a pivotal role in shaping the tumor microenvironment (TME) and driving tumor growth, metastasis, and immune evasion through cell-to-cell communication (1). Cancer cell-derived EVs perform a range of functions and their influence on immune modulation has gained increasing attention. To evade immune detection, cancer cells utilize multiple strategies, and EVs play a crucial role in suppressing immune responses and facilitating immune evasion. EVs promote cancer progression while limiting the efficacy of immune-based therapies by transporting immunosuppressive factors, modulating immune cell activity, and reshaping the TME into an immune-resistant niche. A major concern regarding this phenomenon is the involvement of EVs in inducing resistance to immunotherapy, particularly to immune checkpoint inhibitors (ICIs), such as anti-Programmed Death-1 (PD-1) and anti- Programmed Death-Ligand 1 (PD-L1) antibodies. These therapies aim to restore anti-tumor immunity by reactivating exhausted T cells. However, growing evidence indicates that cancer cell-derived EVs can negatively regulate these responses by carrying immune checkpoint proteins, such as PD-L1, thus contributing to systemic immune suppression (2).

Although immunotherapy has the potential to transform cancer treatment, its overall success is hindered by primary or acquired resistance, which affects a substantial proportion of patients. A deeper understanding of the mechanisms underlying immunotherapy resistance is essential for developing novel therapeutic strategies to improve treatment outcomes. This review explores the role of EVs in cancer biology, emphasizing their involvement in immune evasion and resistance to immunotherapy. Additionally, we discuss potential strategies for counteracting EV-mediated immunosuppression and highlight future directions for enhancing cancer immunotherapy.

## 2 EVs in cancer biology

EVs are increasingly recognized as key mediators of intercellular communication in cancer, influencing tumor progression, immune modulation, metastasis, and therapeutic resistance. These vesicles, secreted by tumor cells, carry a diverse range of bioactive molecules, including proteins, lipids, nucleic acids, and metabolites, which contribute to shaping the TME. EVs contribute to fundamental cancer hallmarks by transferring oncogenic signals between cells, including uncontrolled proliferation, angiogenesis, immune evasion, and metastatic dissemination (1). Elucidation of these molecular mechanisms may enable the development of EV-targeted therapeutics and biomarker-driven strategies for precision oncology.

Cancer cells exploit EVs to enhance their survival and proliferation by transmitting oncogenic factors that support tumor growth and metabolic adaptation. These vesicles carry miRNAs, including *miR-21* and *miR-155*, which regulate pathways involved in cancer cell proliferation, apoptosis, and chemoresistance (3, 4). Additionally, EVs contain metabolic regulators including hexokinase 2 and lactate dehydrogenase, which promote the Warburg effect, a metabolic reprogramming process that enhances glucose uptake and lactate production to sustain rapid tumor growth. This metabolic shift provides cancer cells with a continuous energy supply, while modifying the surrounding environment to favor tumor expansion (5).

As tumors grow, they require an adequate blood supply, and EVs contribute to this process by promoting angiogenesis. Cancer cell-derived EVs carry key angiogenic factors, such as vascular endothelial growth factor, fibroblast growth factor, and hypoxia-inducible factor-1α (HIF-1α), which enhance endothelial cell proliferation and migration, leading to the formation of new blood vessels (6). Additionally, EVs containing *miR-210* suppress antiangiogenic regulators, thereby promoting the angiogenic switch (7). These molecular interactions not only ensure sufficient oxygen and nutrient supply to tumor cells, but also facilitate their infiltration into surrounding tissues (8).

Beyond local tumor progression, EVs play a pivotal role in metastatic dissemination by priming the colonization of distant organs. A key mechanism involves EV-associated integrins α6β4 and α6β1, which guide metastatic cancer cells to specific organs, such as the lungs, liver, and brain (9). In addition, EVs facilitate extracellular matrix remodeling by delivering matrix metalloproteinases that degrade structural barriers, allowing tumor cells to invade and establish secondary tumors. These interactions create a conducive environment for circulating tumor cells to initiate metastasis, ultimately inducing cancer progression to an advanced stage (10).

In addition to inducing tumor growth and metastasis, EVs contribute to evasion of the immune system, allowing cancer cells to escape immune surveillance. This is achieved by impairing T cell function through the induction of exhaustion and reduced cytotoxic activity, ultimately leading to diminished responsiveness to ICIs. Furthermore, EVs promote the expansion of regulatory T-cells (Tregs), contributing to the impairment of anti-tumor immunity by dampening cytotoxic T cell function. Another key immunosuppressive mechanism is the induction of myeloid-derived suppressor cells (MDSCs), which are expanded and activated through EV-mediated signaling, thereby suppressing T cell proliferation and anti-tumor immune responses (11–13). Additionally, EVs impair natural killer (NK) cell activity by downregulating activating receptors required for NK cell-mediated tumor elimination (14). By modulating immune regulatory pathways, EVs facilitate cancer immune evasion and ultimately reduce the efficacy of immune-based therapies.

Given their extensive role in shaping the TME, EVs have emerged as promising therapeutic targets. Efforts to block EV production, neutralize their cargo, or harness them for drug delivery are being actively investigated to counteract their tumor-promoting effects. Moreover, the presence of cancer cell-derived EVs in bodily fluids highlights their potential as biomarkers for cancer diagnosis and treatment response monitoring. Despite advances in our understanding of EV functions, further research is essential to develop effective strategies that can selectively target EV-mediated signaling pathways without disrupting essential physiological processes.

## 3 Mechanisms of EV-mediated immune evasion and immunotherapy resistance

EVs mediate immune suppression, enabling cancer cells to evade immune destruction and resist immunotherapy. By delivering immunomodulatory molecules, EVs impair T-cell function, promote Treg expansion, inhibit NK cell cytotoxicity, and foster an immunosuppressive TME. Moreover, cancer cell-derived EVs carry immune checkpoint proteins that contribute to ICI resistance, thereby diminishing the efficacy of immunotherapy (15). However, one of the most critical mechanisms of EV-mediated immune suppression is the direct inhibition of T cell function. EVs suppress T cell function by delivering PD-L1, FasL, TGF-β, and IL-10, leading to T cell exhaustion and reduced anti-tumor immunity. Although this mechanism resembles PD-L1 expression in tumor cells, PD-L1-containing EVs have a broader impact by systemically circulating and suppressing immune responses at distant sites. Furthermore, EVs deliver FasL, which triggers apoptosis in activated T cells and further reduces anti-tumor immunity (16). By modulating these pathways, EVs establish an immunosuppressive environment that supports tumor survival and progression.

In addition, cancer-derived EVs enhance glycolysis in tumor-associated macrophages and myeloid-derived suppressor cells, further acidifying the TME and impairing effector T cell infiltration (17–19). Hypoxic EVs enriched in HIF-1α promote immune suppression by recruiting immunosuppressive myeloid cells and inhibiting cytotoxic T cell function (20–22). Notably, EVs also promote Treg expansion via TGF-β and IL-10, intensifying immune suppression within the TME. Consequently, EV-mediated Treg expansion contributes to immunotherapy resistance, because excessive Treg activity diminishes the efficacy of immune checkpoint blockade therapy (23). Similarly, cancer cell-derived EVs impair NK cell cytotoxicity via TGF-β, CD73, and FasL, downregulating NKG2D and facilitating immune evasion (24). Moreover, EVs contribute to MDSC expansion via HSP72- and *miR-21*-induced STAT3 activation, further suppressing T-cell responses (25, 26). Additionally, EVs facilitate metabolic reprogramming within the TME by transferring enzymes and metabolites that promote local hypoxia and acidity, creating an environment that is hostile to immune cells, but conducive to tumor progression (27).

A major concern in EV-mediated immune evasion is its contribution to resistance against ICIs, such as anti-PD-1 and anti-PD-L1 antibodies. Although ICIs are designed to restore T-cell function by blocking inhibitory signals, cancer cell-derived EVs act as decoys by carrying PD-L1, thereby neutralizing the therapeutic efficacy of these drugs. As a result, circulating PD-L1^+^ EVs not only suppress T cell activation, but also sequester checkpoint inhibitors, reducing their ability to block tumor-associated PD-L1 (28). Importantly, the role of EVs in immune evasion and therapy resistance may differ depending on tumor type. Notably, their immunosuppressive functions have been more extensively studied in solid tumors, such as melanoma, triple-negative breast cancer (TNBC), and hepatocellular carcinoma, where EVs carry immune checkpoint ligands or immunomodulatory RNAs that suppress anti-tumor immunity (29, 30). In contrast, EVs in hematological malignancies often participate more prominently in altering the bone marrow microenvironment, promoting niche remodeling and facilitating chemoresistance (31, 32). Such tumor-specific variations necessitate the development of more sophisticated and individualized EV-based therapeutic approaches tailored to the distinct biological contexts of each cancer type. Thus, targeting EVs production or their immunosuppressive cargo may enhance the efficacy of immunotherapy.

Emerging approaches include pharmacological inhibition of EVs release, with agents such as GW4869, a neutral sphingomyelinase inhibitor that effectively reduces exosome secretion and tumor-promoting signals (33). In addition, neutralization of immunosuppressive EVs-associated cargo, such as PD-L1, has been shown to restore T-cell activity and enhance responses to ICIs (34). The development of engineered EVs capable of delivering therapeutic agents to specific immune or tumor targets represents another innovative approach (35). Moreover, the identification of EV-derived biomarkers predictive of immunotherapy response holds significant promise for advancing precision oncology, although clinical validation remains an ongoing challenge (36, 37). Collectively, these strategies highlight the therapeutic potential of EV-targeting interventions, but systematic preclinical and clinical validation is needed to translate these findings into clinical practice.

## 4 Cancer cell-derived EVs and immunotherapy resistance

Immunotherapy has emerged as an innovative approach that uses the immune system to treat cancer. However, resistance to immunotherapy remains a significant challenge, and increasing evidence suggests that cancer cell-derived EVs play a pivotal role in this process. In this section, we describe the role of cancer cell-derived EVs in immune suppression and resistance to cancer therapy based on previous studies. These data are summarized in **Table 1**.

**Table 1:** Role of cancer-derived extracellular vesicles in immune suppression and resistance to cancer therapy.

Cancer Type	Cargo loaded in EVs	Description	Reference
Prostate and colon cancer, adenocarcinoma	PD-L1	Act as decoys, accelerating anti-PD-L1 clearance by macrophages	(23)
Breast, colon, and lung cancer	PD-L1	Lower the response to immune checkpoint blockade drugs	(24)
Metastatic melanoma	PD-L1	Suppress the function of CD8^+^ T cells and facilitate tumor growth	(25)
Leukemia	CD19	Induce CAR-T cell exhaustion and reducing cytotoxicity	(26)
Breast cancer	CXCL1	Increase PD-L1 expression levels in tumor-associated macrophages	(27)
Breast cancer	Efflux transporters, miRNAs	Enhance survival, inhibit apoptosis, and suppress immunity.	(28)
Colorectal cancer	–	Induce CD8^+^ T-cell apoptosis and alter cytokine expression	(29)
Liver cancer	HSP90α	Promote IL-6/IL-8 secretion, suppress CD8^+^ T cells, and reduce anti-PD-1/PD-L1 efficacy	(30)
Breast, prostate, renal, ovarian, renal, and bladder cancer, hepatocellular carcinoma	EBAG9	Suppressing T-cell cytotoxicity and modulate immune gene expression	(31)
Breast and ovarian cancer, acute myelogenous leukemia, head and neck squamous cell carcinoma, melanoma	FasL, MHC class I molecules	Induce CD8^+^ T-cell apoptosis	(32)
Pancreatic cancer	*miR-203*	Downregulate TLR4 in dendritic cells, reduce TNF-α and IL-12 production, and suppress DC-mediated immunity.	(33)
Breast and ovarian cancer, acute myelogenous leukemia, head and neck squamous cell carcinoma, melanoma	NKG2D ligands	Suppress NK cell function by downregulating NKG2D expression levels and reducing cytotoxicity	(32)
Pancreatic ductal adenocarcinoma	T and B cell epitopes	Reduce B cell function by triggering autoantibody production and absorbing complement attacks	(34)
Esophageal	LAMP1, MMP9	Suppress CD8^+^ T-cell proliferation by differentiating naive B cells into regulatory B cells	(35)
Hepatocellular carcinoma	HMGB1	Induce CD8^+^ T-cell suppression by expanding Bregs via TLR2/4-MAPK signaling	(36)
Ovarian	*miR-222-3p*	Induce macrophage polarization into the tumor-promoting M2 phenotype via the SOCS3/STAT3 pathway	(37)
Nasopharyngeal carcinoma	*hsa-miR-24-3p*, hsa-miR-891a, *hsa-miR-106a-5p*, *hsa-miR-20a-5p*, and *hsa-miR-1908*	Promote Treg induction, inhibit Th1/Th17 differentiation, and alter T-cell signaling pathways	(38)
Gastric cancer	PD-L1	Enhance MDSC expansion via IL-6/STAT3 signaling	(39)
Glioblastoma	EGFRvIII	Promote tumor growth and resistance to targeted therapies by activating MAPK and Akt pathways	(1)
Gastric and lung cancer, melanoma	TGF-β, Src, Wnt3, HIF1 α	Induce stromal transformation and create a pro-tumor niche	(41)
Pancreatic cancer	CD73	Activate mast cells via adenosine signaling, upregulate pro-angiogenic and tissue-remodeling factors	(42)

The table presents an overview of the roles of cancer-derived EVs in immune suppression and resistance to cancer therapy across various cancer types. It categorizes different cancer types, the specific cargo loaded in EVs, their functional effects on immune cells, and their contribution to tumor progression and therapy resistance. Key molecules such as PD-L1, CXCL1, HSP90α, miRNAs, and FasL are highlighted, demonstrating their impact on T-cell apoptosis, immune evasion, and suppression of NK cell function.

Cancer cell-derived EVs directly contribute to immunotherapy resistance by interfering with ICIs and chimeric antigen receptor (CAR)-T cells. ICIs, such as anti-PD-1/PD-L1 antibodies, are powerful treatments that enhance the ability of the immune system to recognize and attack cancer cells by blocking inhibitory signals that suppress T cell activity. PD-L1 present in cancer cell-derived EVs can contribute to immunotherapy resistance by acting as a decoy that binds to anti-PD-L1 antibodies (38). Tumor-cell-derived EVs carrying PD-L1 also interact with PD-1 on T cells, leading to immune suppression and reduced efficacy of immune checkpoint blockade therapies (39). PD-L1-containing EVs have been shown to correlate with the patient response to anti-PD-1 therapy in metastatic melanoma (37). In addition, cancer cell-derived EVs impair the efficacy of CAR-T cell therapy. EVs containing CD19 interact with CAR-T cells, inducing proinflammatory cytokine secretion and promoting T cell exhaustion. This interaction weakens the therapeutic efficacy and reduces CAR-T cell cytotoxicity (40).

Chemotherapy is widely used for cancer treatment; however, several studies have suggested that cancer cell-derived EVs formed after chemotherapy reduce the efficacy of immunotherapy. Chemotherapy-induced cancer cell-derived EVs increase PD-L1 expression levels in tumor-associated macrophages (TAMs) (41). Similarly, EVs from drug-resistant breast cancer cells deliver efflux transporters and miRNAs that enhance cell survival, inhibit apoptosis, and inhibit immune responses (42). In addition, after chemotherapy, cancer cell-derived EVs induce CD8^+^ T-cell apoptosis and alter cytokine-related gene expression, leading to an immunosuppressive TME that hinders effective anti-tumor immunity (43).

Cancer cell-derived EVs contain various molecules that significantly contribute to immunotherapy resistance by affecting both immune and immunosuppressive cells. For example, cancer cell-derived EVs carrying HSP90-alpha promote the secretion of IL-6 and IL-8 by monocytes and neutrophils, resulting in CD8^+^ T cell suppression and reduced efficacy of anti-PD-1/PD-L1 treatment (44). Cancer cell-derived EVs transfer estrogen receptor-binding fragment-associated antigen 9 to T cells, suppressing cytotoxicity and modulating immune-related gene expression (45). Cancer cell-derived EVs can also induce CD8^+^ T-cell apoptosis via the membrane-associated form of the FasL and MHC class I molecules (46).

Cancer cell-derived EVs suppress the function of other immune cells, including dendritic, NK, and B cells, contributing to immune evasion and tumor progression. Pancreatic-cancer cell-derived EVs transfer *miR-203* to dendritic cells (DCs), leading to TLR4 downregulation and reduced TNF-α and IL-12 production, thereby suppressing DC-mediated immune responses (47). Cancer cell-derived EVs suppress NK cell function by down-regulating NKG2D receptor expression and decreasing cytotoxicity (46). They present tumor antigens to B cells to trigger autoantibody production, while simultaneously acting as decoys that absorb complement attacks, thereby reducing complement-mediated cytotoxicity against cancer cells (48). Cancer cell-derived EVs not only impair B cell function, but also induce their differentiation into regulatory B cells (Bregs), a subset of B cells with immunosuppressive functions that inhibit excessive immune responses and promote immune tolerance. Esophageal cancer cell–derived EVs induce naive B cells to differentiate into Bregs, which suppress CD8^+^ T cell proliferation (49). In hepatocellular carcinoma, Cancer cell-derived exosomes induce the expansion of Bregs via HMGB1-TLR2/4-MAPK signaling, enhancing their ability to suppress CD8^+^ T cell activity (50).

Cancer cell-derived EVs promote the functions of other immunosuppressive cells. Epithelial ovarian cancer cell–derived EVs contain *miR-222-3p*, which regulates the SOCS3/STAT3 pathway and induces macrophage polarization to the tumor-promoting M2 phenotype (51). Cancer cell-derived EVs from nasopharyngeal carcinoma promote Treg induction by inhibiting Th1 and Th17 differentiation and altering T-cell signaling pathways, contributing to immune suppression (52). MDSC expansion can be promoted by cancer cell-derived EVs carrying PD-L1 via IL-6/STAT3 signaling, enhancing immune suppression and tumor progression in gastric cancer (53).

Cancer cell-derived EVs can also act as messengers that facilitate communication between cancer cells, potentially contributing to resistance to immunotherapy. Cancer cell-derived EVs transfer EGFRvIII between tumor cells, activating the MAPK and Akt signaling pathways, which promote tumor growth and metastasis, leading to enhanced resistance to targeted therapies (1). Additionally, EVs facilitate intercellular communication and tumor progression by transferring nucleic acids and proteins between tumor cells and modulating the TME to form a pre-metastatic niche (54).

The TME, which regulates various signals and immunosuppressive cells, significantly influences the efficacy of immunotherapy. For instance, cancer cell-derived EVs containing TGF-β, Src, Wnt3, and HIF1α are taken up by TAMs, which subsequently release membrane blebs, transferring these components to stromal cells (55). Blebs secreted by TAMs carrying cancer cell-derived components induce myofibroblastic changes in the recipient stromal cells, creating a favorable niche for cancer cells (55). Cancer cell-derived EVs activate mast cells through CD73-mediated adenosine signaling, leading to the upregulation of pro-angiogenic and tissue remodeling factors, which contribute to an immunosuppressive TME (56).

Despite significant advances in elucidating the immunosuppressive roles of tumor-derived EVs, emerging evidence points to their dual and context-dependent functions. As demonstrated in preclinical vaccination models, some EVs can stimulate antitumor immunity by delivering tumor-associated antigens and MHC molecules to dendritic cells (57). These contrasting findings highlight the complexity of EVs-mediated immune modulation and indicate the need to identify the molecular and contextual factors determining whether EVs suppress or activate immunity. Moreover, EVs heterogeneity within the tumor microenvironment remains a critical yet underexplored factor. Addressing these challenges through standardization efforts, such as the MISEV guidelines (58), and conducting research towards greater standardization in future studies. While certain EVs-derived signatures show promise as predictive immunotherapy response biomarkers, clinical validation remains incomplete. Addressing these challenges will be crucial for fully harnessing the translational potential of EVs in cancer immunotherapy.

## 5 Conclusions

**Figure 1:**
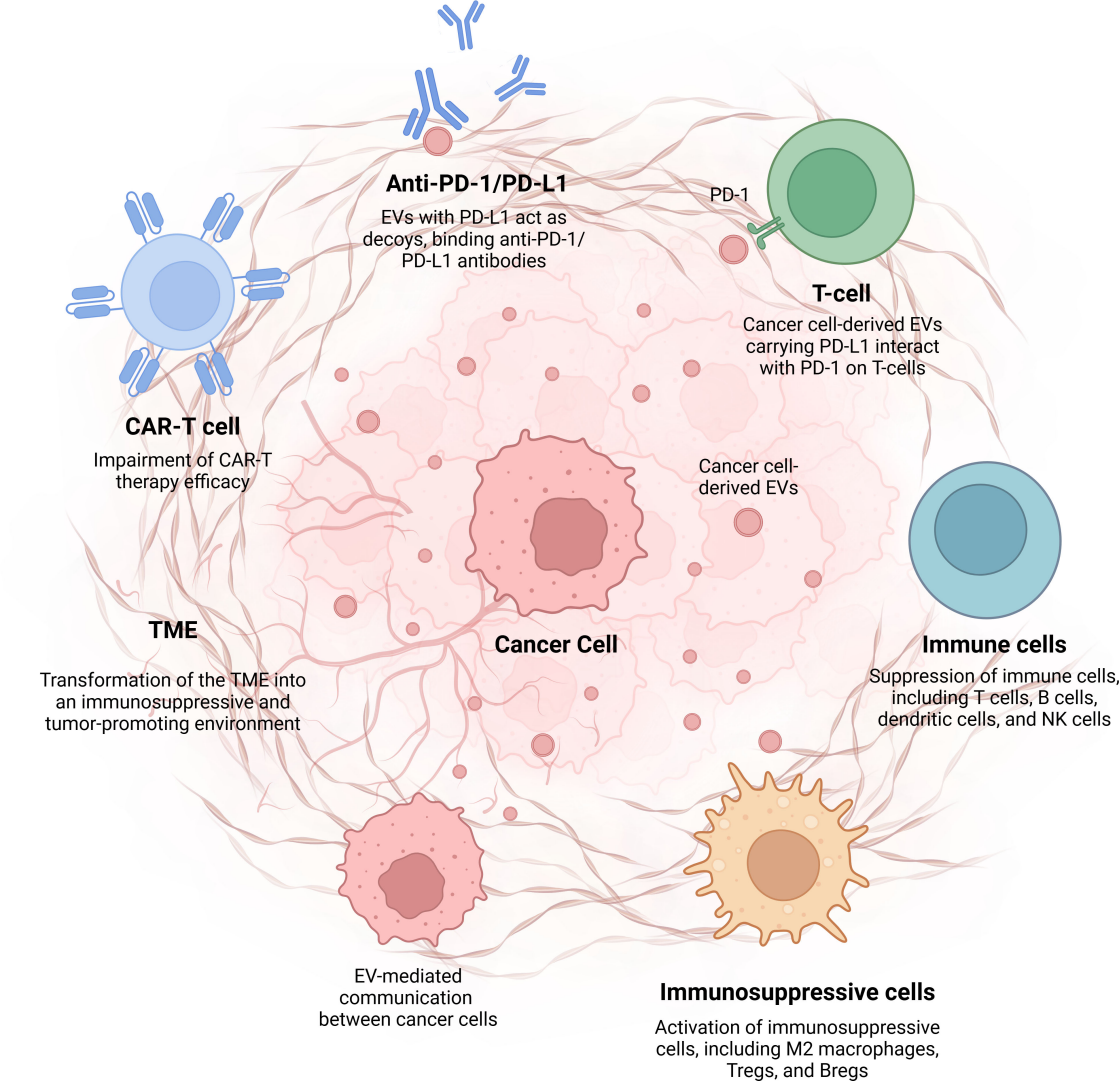
Cancer cell-derived EVs and their role in immunotherapy resistance. This figure illustrates the mechanisms by which cancer cell-derived extracellular vehicles (EVs) contribute to immunotherapy resistance by modulating the tumor microenvironment and suppressing the immune response. EVs, Extracellular Vesicles; PD-LI, Programmed Death-Ligand 1; PD-1, Programmed Death-1; NK, Natural Killer; Tregs, Regulatory T cells; Bregs, Regulatory B cells; TME, Tumor Microenvironment; CAR-T cell, Chimeric Antigen Receptor-T cell. Image was created with BioRender (https://biorender.com/)

Cancer cell-derived EVs play a crucial role in immunotherapy resistance by modulating the immune system and promoting an immunosuppressive TME (**Table 2**). These vesicles carry various immunosuppressive molecules, including PD-L1, TGF-β, and FasL, which impair the function of immune cells such as T cells, NK cells, and DCs. By influencing immune regulation and fostering immune evasion, EVs can contribute to the failure of ICIs and CAR-T cell therapies. Furthermore, EVs facilitate communication between tumor cells, thereby enhancing metastasis and resistance to targeted therapies. Understanding the mechanisms by which EVs contribute to immunotherapy resistance is essential for developing novel therapeutic strategies that can counteract their effects and improve the efficacy of cancer immunotherapies. Targeting EVs is a promising approach to overcome resistance and enhance treatment outcomes in cancer therapy. In this review, we highlighted the critical role of cancer cell-derived EVs in promoting immunotherapy resistance, underscoring the need for targeted strategies to counteract EV-mediated immune evasion and improve therapeutic outcomes (**Figure 1**).

**Table 2:** Overview of immunomodulatory roles and therapeutic implications of cancer-derived EVs.

Category	EV-mediated Mechanisms	Mechanistic Detail
v Scope of EVs Action	♦ Local (Tumor Microenvironment)	- Suppression of cytotoxic T cells - Induction of Tregs and MDSCs - NK cell dysfunction - Promotion of angiogenesis and stromal remodeling
	♦ Systemic (Distal Sites)	- Circulating PD-L1^+^ EVs mediating systemic immunosuppression - Preparation of pre-metastatic niches
v Mechanisms of Immune Modulation	♦ Direct Effects	- Delivery of immunosuppressive molecules - Induction of T cell apoptosis and exhaustion
	♦ Indirect Effects	- Expansion of Tregs and MDSCs - Reprogramming of myeloid and lymphoid compartments
v Targeted Immune Components	♦ Innate Immunity	- Inhibition of NK cell cytotoxicity - Impairment of dendritic cell function
	♦ Adaptive Immunity	- Suppression of effector T cell responses - Expansion of immunoregulatory populations
v Therapeutic Implications	♦ Targetable Processes	- Inhibition of EVs production and release - Neutralizing immunosuppressive cargo - Engineering EVs for therapeutic delivery
	♦ Biomarker Applications	- Development of EVs-derived molecular signatures for predicting immunotherapy outcomes

This table summarizes the immunomodulatory roles of cancer-derived EVs, including their local and systemic effects, mechanisms of immune suppression, targeted immune components, and potential therapeutic and biomarker applications.

Although EV-targeted therapies show promise, several limitations must be considered. Systemic inhibition of EVs may lead to unintended toxicity by interfering with physiological intercellular communication. Moreover, off-target effects remain a concern due to the challenge of distinguishing tumor-derived EVs from those released by normal cells (58). Strategies for selective targeting, such as ligand-directed delivery or nanoparticle engineering, are under development but require further optimization (59, 60).

## 6 Discussion

Concrete bioengineering strategies involve genetic modification of donor cells to enrich EVs with therapeutic cargos (e.g., siRNAs, ICIs), electroporation-based loading methods, and surface functionalization with antibodies or targeting ligands (e.g., anti-EGFR antibodies) (60–63). Preclinical models utilizing engineered EVs have shown promising results in overcoming immune resistance and enhancing the delivery of checkpoint inhibitors (64).

Recent advances in detection technologies such as next-generation sequencing (NGS), nanoplasmonic platforms, and microfluidic enrichment have improved EV analysis by enhancing the sensitivity and specificity of EV-based diagnosis (65–68). For instance, plasma-derived EV RNA signatures have been employed to predict therapy response in non-small cell lung cancer (NSCLC) (69). Several clinical studies have demonstrated the prognostic and predictive value of EV biomarkers, such as PD-L1+ EVs, in lung cancer, melanoma, and colorectal cancer (70–73). Ongoing clinical trials further support these findings, evaluating EV-based biomarkers for treatment response monitoring, minimal residual disease detection, and early cancer diagnosis across various tumor types such as lung cancer, breast cancer, pancreatic cancer, and colorectal cancer (74).

Despite the growing interest in EVs as key players in cancer progression, several controversies and unresolved challenges remain. A major issue is their heterogeneity and the classification of EVs to distinguish them from exosomes, microvesicles, and other subtypes remains unclear, because of overlapping size ranges and the absence of universally accepted markers (75). This complicates the standardization of EV isolation and characterization, leading to inconsistencies across studies (76). Additionally, although cancer cell-derived EVs carry oncogenic cargo, distinguishing them from normal-cell-derived EVs remains difficult, limiting their diagnostic potential (77–79).

Beyond tumor progression, cancer cell-derived EVs critically contribute to immune evasion and resistance to immunotherapy. ICI therapies, such as anti-PD-1/PD-L1 therapies, can induce the release of PD-L1^+^ EVs, which act as decoys to neutralize therapeutic antibodies and suppress T-cell responses (80). Similarly, tumors treated with CAR-T cells secrete EVs carrying inhibitory molecules that induce T cell exhaustion (81). Furthermore, chemotherapy-induced EVs can enhance PD-L1 expression levels in TAMs, creating an immunosuppressive TME (82). These EVs also carry immunosuppressive molecules, including PD-L1, TGF-β, and IL-10, which inhibit T cell activation or promote components of immune suppressive TME, such as Tregs and MDSCs, further dampening anti-tumor immunity (83, 84). The ability of EVs to establish an immunosuppressive microenvironment directly contributes to tumor immune escape and reduces the efficacy of ICIs.

To fully exploit EVs in cancer diagnosis and therapy, future research should focus on standardizing high-throughput EV isolation techniques (85) and utilizing advanced analytical tools, such as super-resolution microscopy and microfluidics-based platforms, to better understand EV heterogeneity (86). Additionally, engineered EVs are being explored as therapeutic vesicles using approaches such as selectively loading EVs with ICIs or small interfering RNAs to counteract tumor-induced immune suppression (87).

Bridging the gap between EV research and clinical applications requires collaborations among researchers, clinicians, and engineers. Large-scale clinical trials are needed to validate EV-based biomarkers and therapies for regulatory approval and widespread adoption (88). PD-L1-expressing EVs are emerging predictive biomarkers of ICI efficacy, enabling patient stratification for personalized treatment (89). Engineered EVs carrying immunomodulatory agents may offer novel strategies to overcome immune resistance (90). These emerging applications emphasize the need for further research on EVs as mediators and therapeutic targets in immunotherapeutic resistance.
